# Overexpression of *AtAHL20* causes delayed flowering in Arabidopsis via repression of *FT* expression

**DOI:** 10.1186/s12870-020-02733-5

**Published:** 2020-12-11

**Authors:** Reuben Tayengwa, Pushpa Sharma Koirala, Courtney F. Pierce, Breanna E. Werner, Michael M. Neff

**Affiliations:** 1 Program in Molecular Plant Sciences, Washington State University, Pullman, WA 99164 USA; 2 Department Crop and Soil Sciences, Washington State University, Pullman, WA 99164 USA; 3 Present address: Plant Sciences and Horticultural Landscape Department, University of Maryland, College Park, MD 20742 USA; 4 Present address: Washington State Department of Fish and Wildlife, Olympia, WA 987501 USA; 5 Present address: United States Department of Agriculture, Animal and Plant Health Inspection Service, Wildlife Services, National Wildlife Research Center, Fort Collins, CO 80521 USA; 6 Present address: Washington State University College of Nursing, Spokane, WA 99202 USA

**Keywords:** *AHL*, *AHL20*, Arabidopsis, AT-hook, Flowering, *FT*

## Abstract

**Background:**

The 29-member Arabidopsis *AHL* gene family is classified into three main classes based on nucleotide and protein sequence evolutionary differences. These differences include the presence or absence of introns, type and/or number of conserved AT-hook and PPC domains. *AHL* gene family members are divided into two phylogenetic clades, Clade-A and Clade-B. A majority of the 29 members remain functionally uncharacterized. Furthermore, the biological significance of the DNA and peptide sequence diversity, observed in the conserved motifs and domains found in the different AHL types, is a subject area that remains largely unexplored.

**Results:**

Transgenic plants overexpressing *AtAHL20* flowered later than the wild type under both short and long days. Transcript accumulation analyses showed that *35S:AtAHL20* plants contained reduced *FT, TSF, AGL8* and *SPL3* mRNA levels. Similarly, overexpression of *AtAHL20’s* orthologue in *Camelina sativa,* Arabidopsis’ closely related *Brassicaceae* family member species, conferred a late-flowering phenotype via suppression of *CsFT* expression. However, overexpression of an aberrant *AtAHL20* gene harboring a missense mutation in the AT-hook domain’s highly conserved R-G-R core motif abolished the late-flowering phenotype. Data from targeted yeast-two-hybrid assays showed that AtAHL20 interacted with itself and several other Clade-A Type-I AHLs which have been previously implicated in flowering-time regulation: AtAHL19, AtAHL22 and AtAHL29.

**Conclusion:**

We showed via gain-of-function analysis that *AtAHL20* is a negative regulator of *FT* expression, as well as other downstream flowering time regulating genes. A similar outcome in *Camelina sativa* transgenic plants overexpressing *CsAHL20* suggest that this is a conserved function. Our results demonstrate that *AtAHL20* acts as a photoperiod-independent negative regulator of transition to flowering.

**Supplementary Information:**

The online version contains supplementary material available at 10.1186/s12870-020-02733-5.

## Background

The 29-member Arabidopsis *AT-HOOK MOTIF CONTAINING NUCLEAR LOCALIZED (AHL)* gene family is found in all sequenced plant species, ranging from the moss *Physcomitrella patens* to flowering plants such as *Arabidopsis thaliana*, *Sorghum bicolor*, *Zea mays* and *Populus trichocarpa* [49, 50]. AHL proteins are characterized by two conserved structural units: the AT-hook motif and the PLANT AND PROKARYOTE CONSERVED (PPC) domain [1, 12].

The AT-hook is a small DNA-binding protein domain which was first characterized in the HIGH MOBILITY GROUP (HMG) non-histone chromosomal protein HMG-I(Y), AHL homologues in mammals [1]. Arabidopsis AHLs contain a conserved arginine-glycine-arginine-proline (R-G-R-P) core motif in the AT-hook domain [35, 49, 50]. When AT-hook domain sequences from all sequenced land plant species are aligned, only arginine-glycine-arginine (R-G-R) amino acid residues remain 100% conserved, suggesting that they are important for function [50]. Studies in HMG proteins and AT-hook motif-containing peptides showed that the AT-hook domain binds to the minor groove of AT-rich DNA via the R-G-R motif [1, 18, 32, 33]. Mutations in this core motif have been shown to abolish DNA binding as well as AHL protein function [14, 17, 35, 47, 49].

Arabidopsis *AHLs* evolved into two major phylogenetic clades: Clade-A and Clade-B [49]. Clade-A and Clade-B *AHLs* underwent multiple gene duplication events which resulted in the expansion of the gene family [50]. Functional characterization of single and multiple gene loss-of-function mutants suggest that genetic redundancy exists among multiple *AHL* genes in Arabidopsis [35, 40, 49]. In previous *AHL* gene knockout studies, single *T-DNA* insertion mutants including *ahl22-1* [40], *sob3-4* and *esc-8* [35] did not show obvious phenotypes, unless other closely related family members were also knocked out. Zhao et al. [49] reported that when specific *AHL* genes were knocked out in higher order combinations, such as in the quadruple *sob3-4 esc-8 ahl6 ahl22,* the resultant plants showed more dramatic phenotypes compared to lower order gene knockout mutant combinations. Furthermore, Zhao et al. [49] also showed that *sob3-6*, a dominant negative mutant carrying a missense allele in the R-G-R core of the AT-hook motif, displayed more dramatic hypocotyl phenotypes compared to the *sob3-4 esc-8 ahl6 ahl22* quadruple mutant. Based on these data, a molecular model was proposed where AHLs interact with each other and themselves, as well as other nuclear proteins, such as transcription factors (TFs), to form a “DNA-AHL-TF complex” [49]. Overall, these data suggest that genetic redundancy exists among *AHLs.* It is hypothesized that most AHLs function as complexes, and that mutations in the DNA-binding AT-hook motif may render that entire complex non-functional [49].

Both loss-of-function and gain-of-function studies in Arabidopsis have demonstrated a role for *AHLs* in plant growth and developmental processes, including auxin, brassinosteroid and gibberellic acid signaling, hypocotyl elongation, petiole growth, root system architecture, environmental stress responses, vascular tissue development, floral organ initiation, organ size, flowering time, and pollen wall development, [7–9, 13, 19, 20, 23, 26, 29, 34, 35, 39–42, 47, 51, 52]. Out of 29 Arabidopsis *AHL* gene family members*,* 13 have been characterized; *AtAHL1, AtAHL3, AtAHL4, AtAHL10, AtAHL15, AHL16, AtAHL18, AtAHL19, AtAHL20, AtAHL22, AtAHL25, AtAHL27* and *AtAHL29* [12, 19, 28, 29, 34, 35, 39, 40, 43, 47, 53]. Interestingly, several of the functionally characterized *AHLs* (*AtAHL16, AtAHL18, AtAHL22, AtAHL27* and *AtAHL29*) have been directly or indirectly implicated in the regulation of flowering time in a redundant manner [40–42, 47]. *AtAHL16, AtAHL18, AtAHL22, AtAHL27* and *AtAHL29* are all Clade-A *AHLs* [49].

In this study, we used a gain-of-function analysis strategy to avoid potential issues associated with genetic redundancy to characterize *AtAHL20 (AT4G14465),* a Clade-A *AHL*. *AtAHL20* was initially selected for functional analysis together with *AtAHL6* (Clade-B *AHL* gene family member) as part of a comparative functional characterization study between *AHLs* that belong to two distinct phylogenetic clades and contain different AT-hook domain types. That work is ongoing; therefore, this study only focuses on *AtAHL20*. Transgenic plants overexpressing *AtAHL20* flowered later than the wild type in long-day (LD) and short-day (SD) conditions. Transcript abundance analysis of the key flowering time regulator, *FLOWERING LOCUS T (FT),* showed that its expression was repressed in *35S:AtAHL20* plants. In addition, *FT’s* redundant homologue, *TSF*, and other downstream flowering pathway genes, *AGL8* and *SPL3,* were also repressed in *35S:AtAHL20* plants. We demonstrated that the second arginine residue in the conserved R-G-R core motif in the AT-hook domain was important for the manifestation of *AtAHL20’s* overexpression phenotypes. Targeted yeast-two hybrid assay results showed that AtAHL20 interacted with itself, its closest family member AtAHL19, as well as other Clade-A AHLs, AtAHL22 and AtAHL29, which have previously been shown to regulate flowering time. Overall, we demonstrated that *AtAHL20* is a photoperiod-independent repressor of *FT* expression and other downstream flowering pathway genes.

## Results

### *AtAHL20* tissue expression pattern

**Fig. 1:**
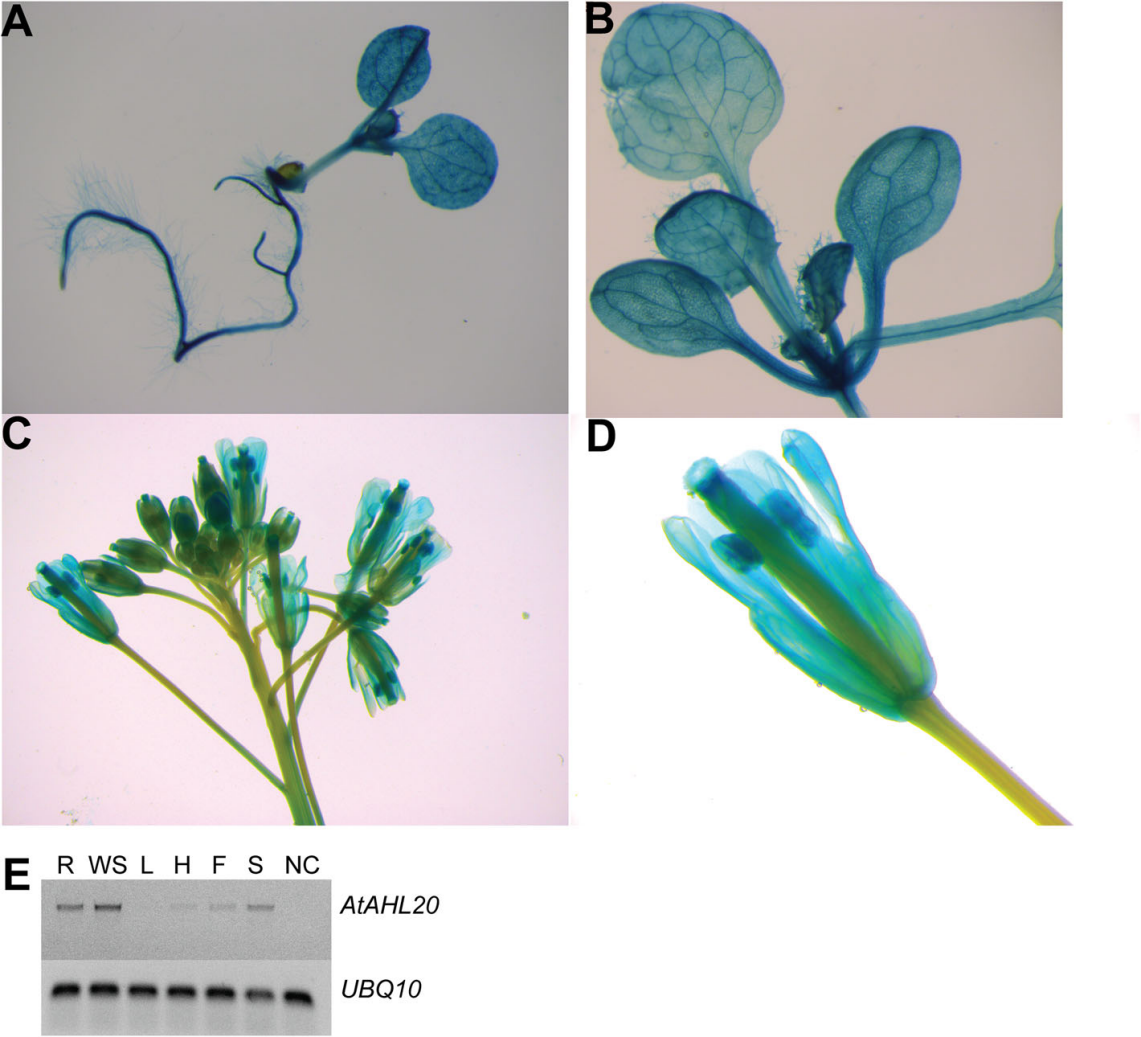
*AtAHL20* promoter-GUS expression pattern. Wild-type Arabidopsis plants were transformed with a construct encoding a *pAtAHL20-GUS* transcriptional fusion. Histochemical analysis was conducted on 6-day old seedlings grown on Murashige and Skoog (MS) agar plates under continuous white light, rosette leaves from 12-day old plants and floral structures of adult plants grown under LD conditions in the greenhouse. GUS histochemical staining patterns in (**a**), 6-day old *pAtAHL20:GUS* seedlings (**b**), rosette leaves from a 12-day old *pAtAHL20:GUS* plant showing expression in minor veins (**c**), *pAtAHL20:GUS* floral structure (**d**), close-up of *pAtAHL20:GUS* floral structure. GUS activity assays were performed three times with similar results. (**e**) Semi-quantitative PCR gel showing *AtAHL20* tissue expression pattern in 7-day old seedling roots (R), 7-day old whole seedling (WS), adult flowering plant rosette leaf (L), 7-day old seedling hypocotyls (H), flower (F), siliques (S) and no reverse transcriptase negative control (NC). *UBQ10* was used as a house keeping gene control

We analyzed *AtAHL20’s* expression pattern via a transcriptional fusion with the *β*-glucuronidase *(GUS)* reporter gene. Strong global *GUS* signal was observed in all tissues, including root hairs, suggesting that *AtAHL20* is constitutively expressed in seedlings (Fig. 1a). 12-day old *pAtAHL20-GUS* transgenic plants displayed *GUS* activity in leaf minor veins and trichomes (Fig. 1b). In floral structures, *GUS* activity was detected in petals, petal vasculature, anthers, stigma and the upper part of the style, but the signal was weaker in the pedicel and peduncle vasculature (Fig. 1c, d). Semi-quantitative PCR analysis also showed differential *AtAHL20* tissue expression pattern in whole seedlings, seedling roots, hypocotyls, flowers and siliques, a trend similar to GUS histochemical staining pattern (Fig. 1e). Largely, *AtAHL20* showed a ubiquitous tissue expression pattern and overlaps with *pFT-GUS* expression pattern in minor veins of young rosette leaves [36].

### *35S:AtAHL20* plants flowered later than the wild type

**Fig. 2:**
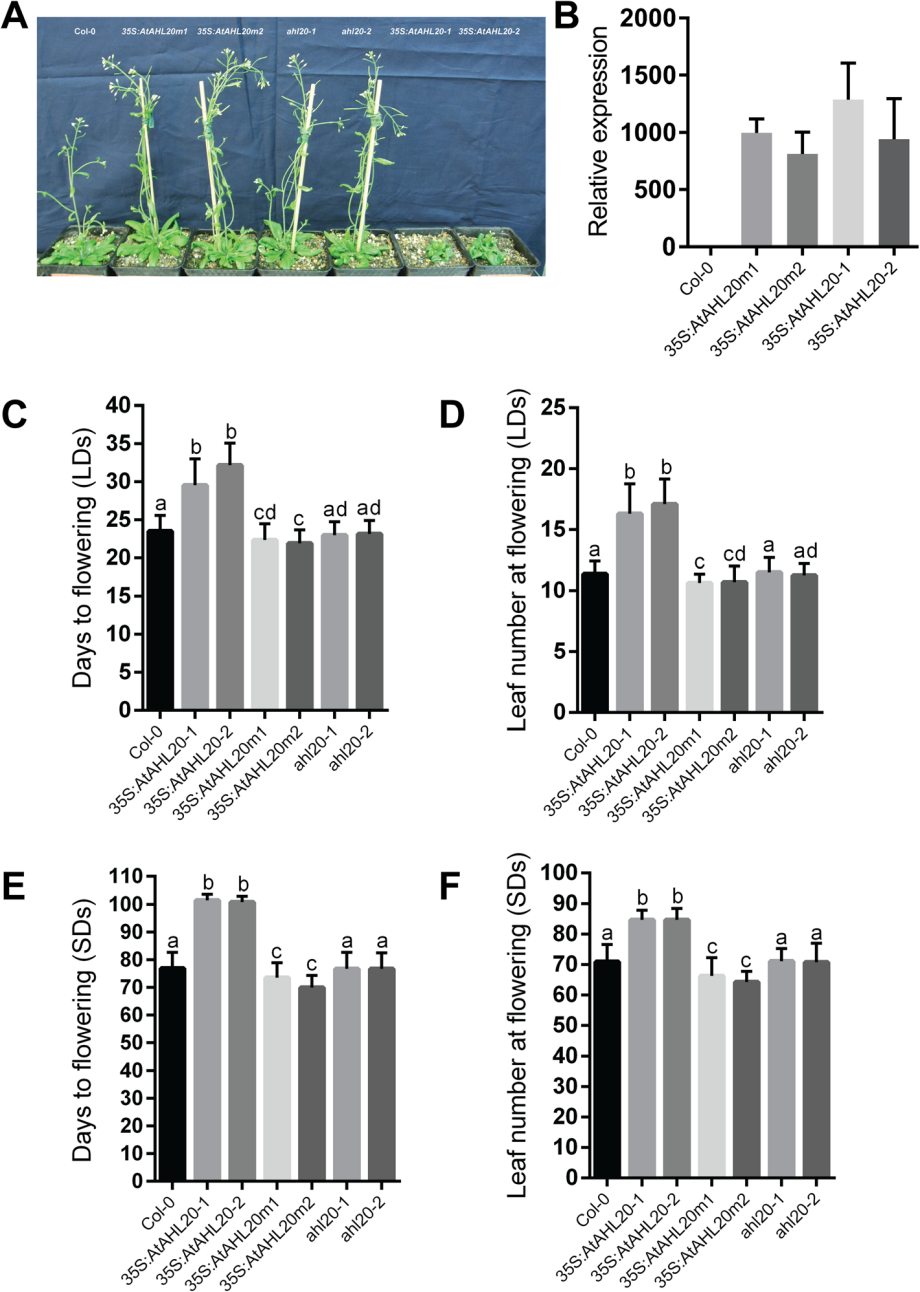
Phenotypic and flowering-time analysis of *35S:AtAHL20* transgenic plants. **a** *35S:AtAHL20* transgenic plants displayed dwarf phenotypes compared to the wild type, *35S:AtAHL20m1, 35S:AtAHL20m2, ahl20-1* and *ahl20-2* plants under LD conditions. **b** *AtAHL20* expression levels in *35S:AtAHL20* and *35S:AtAHL20m* plants. **c** and **d** *35S:AtAHL20-1* and *35S:AtAHL20-2* transgenic plants flowered later than the wild type, *35S:AtAHL20m1,35S:AtAHL20m2, ahl20-1* and *ahl20-2* plants under LD conditions. **e** and **f** *35S:AtAHL20-1* and *35S:AtAHL20-2* transgenic plants flowered later than the wild type, *35S:AtAHL20m1, 35S:AtAHL20m2, ahl20-1* and *ahl20-2* plants under SD conditions. When we overexpressed AtAHL20 protein carrying a point mutation in a conserved R-G-R core motif, the resultant transgenic plants flowered earlier than the wild type (**c**-**f**). Flowering time was calculated by counting the number days from sowing until the appearance of a 1 cm long primary bolt, as well as by counting the total number of primary rosette and cauline leaves present at bolting. The error bar denotes standard deviation (SD). Different letters indicate statistical significance (ANOVA; *P* < 0.05). n = at least 35 plants (between 35 and 48 plants per genotype). The experiment was repeated three times with similar outcomes

Gene overexpression studies and activation-tagging screens have been key tools used to characterize *AHL* gene function [28, 35, 40, 43]. Multiple independent transgenic plants overexpressing *AtAHL20* displayed a dwarf phenotype (Fig. 2a, b) and a late-flowering phenotype compared to the wild type and *ahl20-1*/*ahl20-2 T-DNA* insertion lines under both LDs (Fig. 2c, d) and SDs (Fig. 2e, f). Previously, a conserved arginine amino acid residue in the AT-hook domain was shown to be necessary for *AtAHL22* and *AtAHL29’s* gain-of-function phenotypes [35, 47, 49]. Therefore, we next examined whether this was the case for *AtAHL20*. We generated constructs carrying *AtAHL20* coding sequence harboring a point mutation (*AtAHL20m*) in the conserved R-G-R core motif, changing arginine residue number 72 to a histidine (R72 > H). The resultant *35S:AtAHL20m* transgenic plants overexpressing the mutant protein lost both the dwarf and late-flowering phenotypes observed in *35S:AtAHL20* plants (Fig. 2a-f). Instead, the *35S:AtAHL20m* transgenic plants displayed an early-flowering phenotype compared to wild-type and *T-DNA* insertion lines under both SDs and LDs (Fig. 2c-f) suggesting that *AtAHL20* is a photoperiod-independent floral repressor. These results imply that the second conserved arginine residue is required for the manifestation of *AtAHL20’s* overexpression phenotypes.

### Conserved function of Arabidopsis and Camelina *AHL20* orthologues

**Fig. 3:**
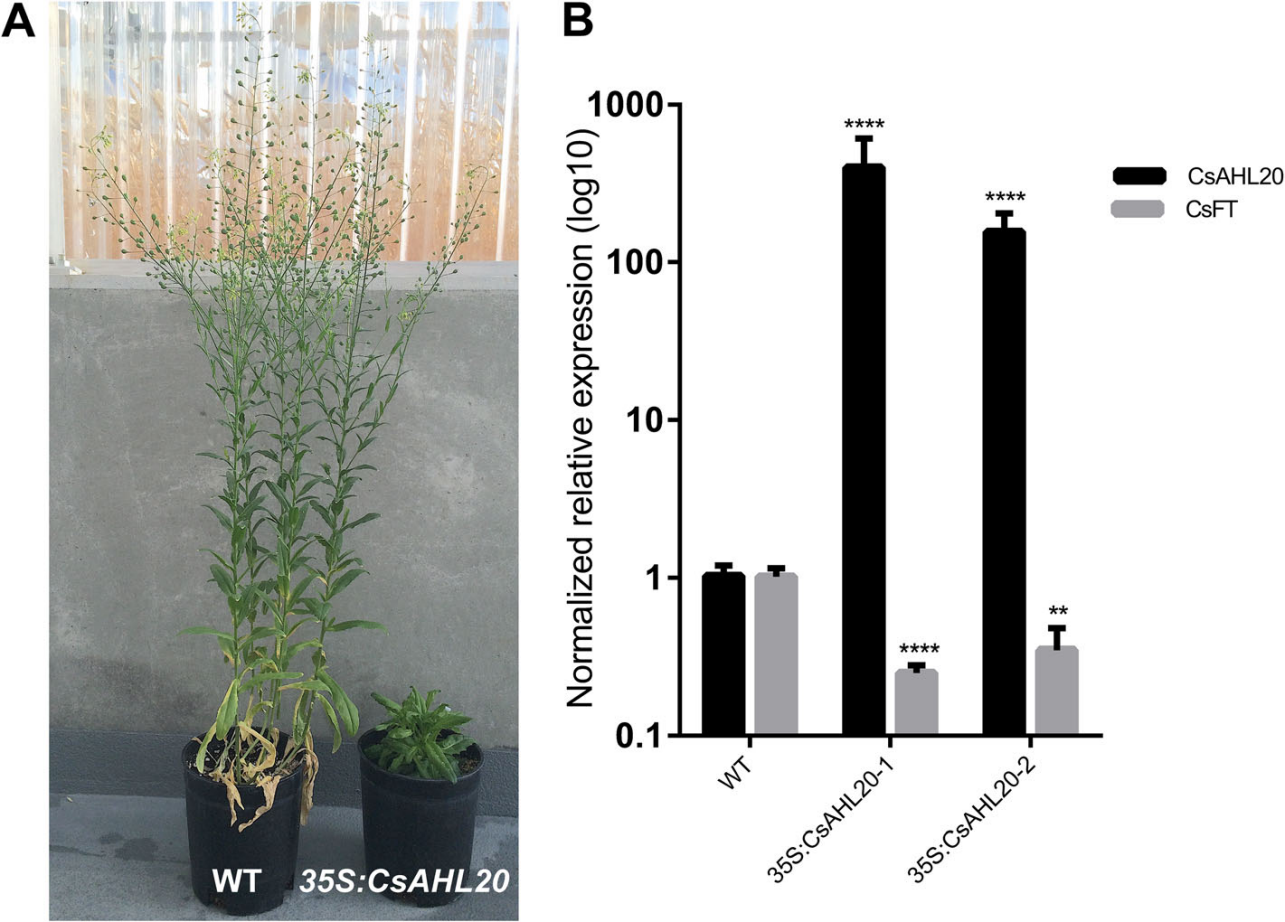
*CsAHL20* overexpression represses *CsFT* expression in *Camelina sativa.* **a** *35S:CsAHL20* plants displayed a dwarf and late-flowering phenotype compared to the wild type. **b** Overexpression of *CsAHL20* in *Camelina sativa* wild-type plants resulted in suppressed *CsFT* transcript levels. T-test statistical analysis was performed using GraphPad Prism software. **** = *p*-value < 0.0001, *** = *p*-value 0.0001 to 0.001, ** = *p*-value 0.001 to 0.01, * = *p*-value 0.01 to 0.05. 3 biological replicates were analyzed (*n* = 3)

Since Arabidopsis and *Camelina sativa* are closely related *Brassicaceae* family member species [2, 21], we hypothesized that some *AHL* orthologues would share similar or overlapping biological functions. To test this hypothesis, one of three Camelina *AHL20-like* copies *CsAHL20 (LOC104718987),* which has high similarity to *AtAHL20* at the nucleotide and protein sequence level was cloned into a binary vector under the *35S CaMV* promoter. The resultant construct was used to transform *Camelina sativa* (L.) Crantz var. Calena wild-type plants. We isolated multiple T_3_ homozygous single-locus insertion overexpression lines, which exhibited late-flowering and dwarf phenotypes compared to wild-type controls (Fig. 3a). These phenotypes were similar to those observed in *35S:AtAHL20* transgenic plants, inferring that Camelina and Arabidopsis *AHL20* genes have similar biological functions.

### *AtAHL20* represses *FT* expression

**Fig. 4:**
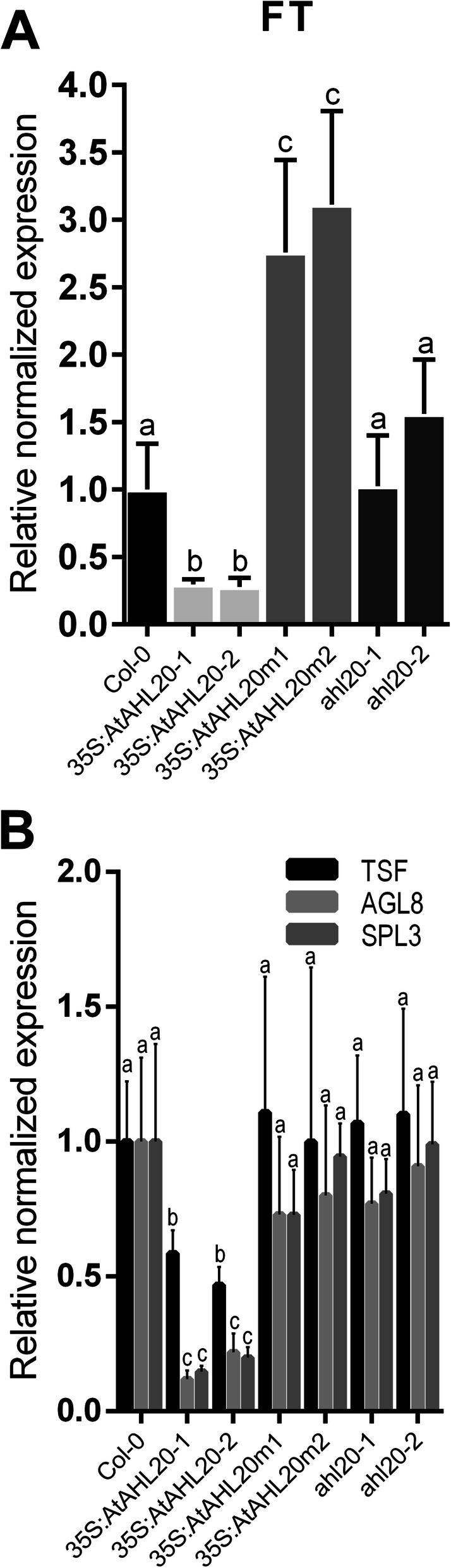
*AtAHL20* overexpression represses *FT* expression under LD conditions. **a** *FT* transcript levels were suppressed to ~ 30% of wild-type levels in *35S:AtAHL20-1* and *35S:AtAHL20-2* plants. In contrast, *FT* transcript abundance was elevated in the dominant-negative mutant plants *35S:AtAHL20m1* and *35S:AtAHL20m2*, but were unchanged in *ahl20-1* and *ahl20-2* T-DNA insertion lines. **b** RT-qPCR analysis data showed that *AGL8*, *SPL3* and *TSF* transcript levels were repressed in *35S:AtAHL20* plants but were unchanged in the wild type and *35S:AtAHL20m1, 35S:AtAHL20m2, ahl20-1* and *ahl20-2* plants. RT-qPCR analysis was performed using RNA extracted from rosette leaves of three biological replicates of 21-day old plants grown under LD conditions. The error bar denotes standard error of mean (SEM). Different letters indicate statistical significance (ANOVA; *P* < 0.05)

To further investigate the cause of the late flowering-time phenotype observed in *35S:AtAHL20* transgenic plants, we measured transcript levels of the key regulatory flowering gene, *FLOWERING LOCUS T (FT)* [4] via reverse transcription quantitative polymerase chain reaction (RT-qPCR). *FT* transcript levels in *35S:AtAHL20* transgenic plants dropped to ~ 30% of wild-type levels (Fig. 4a). This result was similar to that reported in plants overexpressing another Clade-A *AHL* gene family member, *AtAHL22* [40]. In contrast, *35S:AtAHL20m* plants contained elevated *FT* levels compared to both wild-type and *35S:AtAHL20* plants (Fig. 4a), which is consistent with the early-flowering phenotype observed in these plants.

Since *35S:CsAHL20* transgenic Camelina plants displayed a late-flowering phenotype compared to the wild type (Fig. 3a), we hypothesized that this was due to suppression of *CsFT* expression. RT-qPCR data showed that transcript levels of one of three *CsFT* genes in *35S:CsAHL20* plants were repressed four-fold compared to wild-type plants (Fig. 3b). Sequence alignment revealed high similarity between *AtFT* and *CsFT* nucleotide and peptide sequences.

Transcriptional profiling using high throughput next-generation ribonucleic acid (RNA) sequencing (RNA-Seq) is a valuable tool to identify differentially expressed genes on a global level [45, 46]. To gain further insights into the overall flowering-time pathway transcriptome perturbations in *35S:AtAHL20* transgenic plants compared to wild-type plants, we performed RNA-Seq analysis. Kal’s Z-test was performed to identify differentially expressed genes between the wild type and *35S:AtAHL20* transgenic plants (Data S1–2). We identified 1628 downregulated and 2179 upregulated genes in *35S:AtAHL20* transgenic plants compared to the wild type. Gene ontology (GO) analysis [30] was performed based on the down-regulated gene list (Data S1) in *35S:AtAHL20* plants compared to the wild type. This led to the identification of three flowering time regulating genes in a small enriched subset of reproductive development GO terms; *AGAMOUS-LIKE 8 (AGL8/AT5G60910), SQUAMOSA PROMOTER BINDING PROTEIN-LIKE 3 (SPL3/AT2G33810)* and *TWIN SISTER OF FT (TSF/AT4G20370)*. This result was confirmed via RT-qPCR analysis, which showed repression of all three genes in *35S:AtAHL20* plants compared to the wild type (Fig. 4b). However, *AGL8*, *SPL3* and *TSF* transcript accumulation levels were unchanged in *35S:AtAHL20m* and *ahl20* T-DNA insertion mutant plants (Fig. 4b).

### AtAHL20 interacts with other Clade-A AHLs implicated in flowering time regulation

**Fig. 5:**
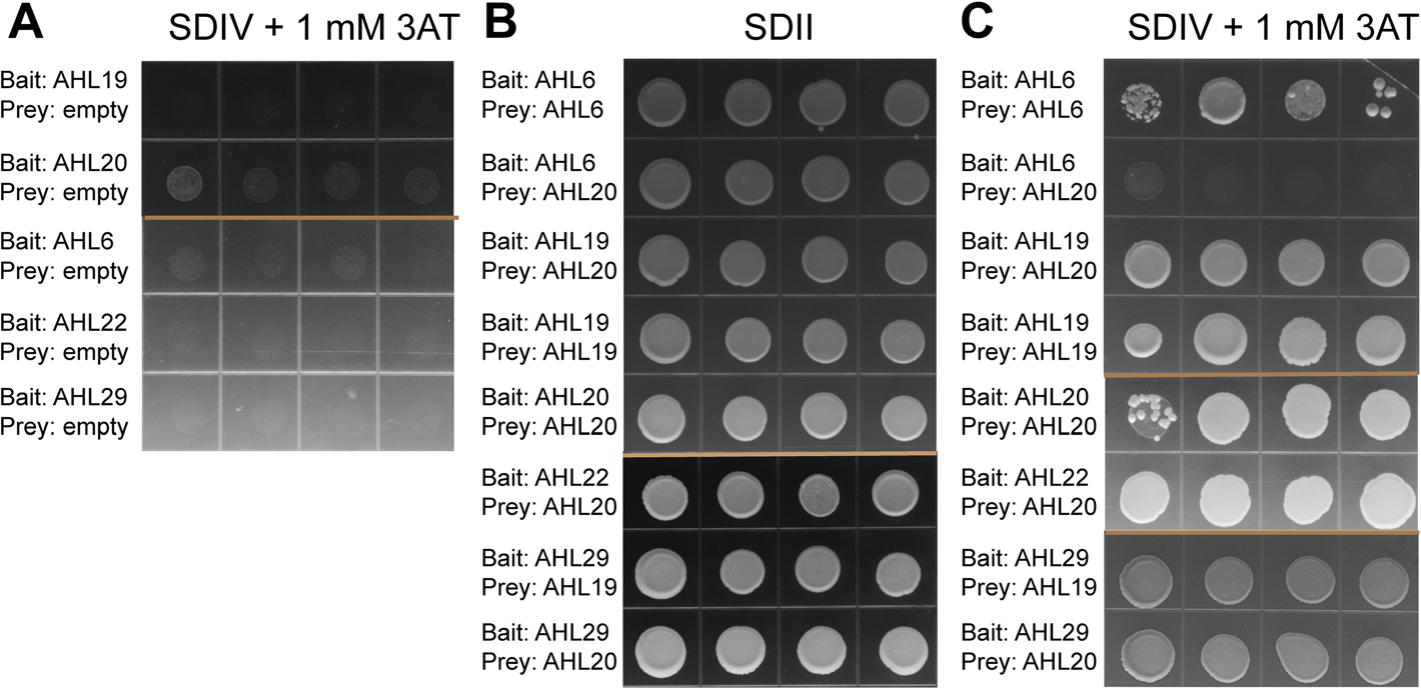
Targeted yeast-two-hybrid assays. **a** Targeted yeast hybrid assays were performed between AtAHL20, AtAHL6, AtAHL19, AtAHL22 and AtAHL29. 1 mM 3-amino-1,2,4-triazole (3-AT) was used to suppress auto-activation of bait proteins. **b** Yeast cells that were co-transformed with both the bait and prey constructs were plated on synthetic dropout II (SDII) media, which lacked tryptophan and leucine, as positive controls to demonstrate successful transformation. **c** Four individual colonies were picked and plated on synthetic dropout IV (SDIV) media lacking tryptophan, leucine, histidine, and uracil but supplemented with 1 mM 3-AT to suppress autoactivation. Clade-B member AtAHL6 and Clade-A member AtAHL20 physically interacted with themselves but not with each other. AtAHL20 and its closest family member AtAHL19 physically interacted with each other, as well as themselves. AtAHL20 also interacted with other Clade-A AHLs: AtAHL22 and AtAHL29. These results may suggest that AHLs function redundantly to regulate flowering time, possibly as part of homo-, hetero-dimers or multiple AHLs protein complex that includes AtAHL19 AtAHL20, AtAHL22, and AtAHL29. AtAHL6, a Clade-B member, was used as a control to show that not all AHLs interacted with each other. Light brown lines show demarcation between panels of Y2H assays that were performed separately, but have been pasted together to make this figure

A few AHL proteins have been shown to interact with themselves and other non-AHL proteins [25, 28, 35, 47, 49]. Interestingly, several Clade-A *AHLs* have been associated with flowering time phenotypes, suggesting that genetic redundancy exists among these genes [40–42, 47]. Therefore, we tested whether any Clade-A AHLs formed homo- and/or heterodimers via targeted yeast-two-hybrid (Y2H) assays. To avoid false positive protein-protein interactions, yeast transformed with bait protein constructs were plated on synthetic defined (SD) media supplemented with a predetermined inhibitory concentration of 1 mM 3-amino-1,2,4-triazole (3-AT) (Fig. 5a). Successful co-transformation of yeast with the two bait and prey protein constructs was demonstrated by growth on SDII media (Fig. 5b). We showed that AtAHL20 interacted with itself to form a homodimer (Fig. 5c). Next, we tested whether other Clade-A AHLs that have been implicated in flowering time regulation interacted with each other to form heterodimers. Indeed, AtAHL20 interacted with AtAHL19, AtAHL22 and AtAHL29. We further asked whether it was possible that all AHLs interacted with each other and tested for interaction between a Clade-B member AtAHL6, and a Clade-A member AtAHL20. There was no interaction between AtAHL6 and AtAHL20, indicating that not all AHLs interact with each other (Fig. 5c, Table 1).

**Table 1:** Yeast two hybrid interactions

Clade	Bait	Interacting partner	Interaction
A/A	AtAHL19	AtAHL19	Positive
A/A	AtAHL20	AtAHL20	Positive
A/A	AtAHL20	AtAHL19	Positive
A/A	AtAHL22	AtAHL20	Positive
A/A	AtAHL29	AtAHL20	Positive
A/A	AtAHL29	AtAHL19	Positive
B/B	AtAHL6	AtAHL6	Positive
B/A	AtAHL6	AtAHL20	Negative

## Discussion

### Overexpression of *AtAHL20* confers a late-flowering time phenotype in Arabidopsis

Our gain-of-function study showed that overexpression of *AtAHL20* confers a late-flowering time phenotype under both SDs and LDs (Fig. 2). This result is consistent with previous work implicating several Clade-A Arabidopsis AHLs (*AtAHL18, AtAHL*22, *AtAHL27* and *AtAHL29)* in flowering time regulation [35, 40, 47]. Specifically, transgenic plants overexpressing *AtAHL22*, *AtAHL27* and *AtAHL29* displayed a late-flowering phenotype [35, 40, 47]. *AtAHL22, AtAHL27* and *AtAHL29* single gene knockout mutants did not show any clear flowering-time phenotypes. Only when *AtAHL27, AtAHL29, AtAHL22* and *AtAHL18* were simultaneously knocked out and/or knocked down, did the quadruple mutant display an early-flowering phenotype [35, 40, 47]. These data suggested that several *AHLs* may function as part of a complex(es) to regulate gene expression [31, 37, 47] and that functional redundancy exists between these genes and other Clade-A *AHL* family members. Indeed, Zhao et al. [49] proposed a similar model suggesting that various AHLs formed multi-AHL complexes to regulate hypocotyl growth in Arabidopsis. Data from our targeted yeast-two-hybrid assays supports this model by demonstrating that AtAHL20 physically interacted with itself and other Clade-A AHL members; AtAHL19, AtAHL22 and AtAHL29 (Fig. 5). It is, therefore, conceivable that these AHLs regulate flowering time as part of a complex. AtAHL20 did not interact with AtAHL6 (a Clade-B *AHL*) indicating that not all AHLs interacted with each other. Overall, we have shown that *AtAHL20* is the fifth Clade-A *AHL* to be implicated in flowering time regulation in Arabidopsis.

### *AtAHL20* is a repressor of *FT* expression

Gene expression analyses showed that overexpression of *AtAHL20* resulted in depletion of *FT* transcript levels (Fig. 4). This is not surprising considering that several *AHLs*, including *OsAHL1*, *AtAHL5, AtAHL10, AtAHL12*, *AtAHL16*, *AtAHL20*, *AtAHL22*, *AtAHL27* and *AtAHL29,* have been reported to exhibit promoter binding capabilities or been shown to confer transcriptional repression or activation of downstream target genes [7, 8, 10, 11, 19, 24, 39, 41, 42, 54]. In particular, a previous study showed that *AtAHL20* is a negative regulator of defenses in Arabidopsis [28]. We also showed that an AHL20 orthologue in Camelina repressed *CsFT* expression, which suggests a conserved function across the two species (Fig. 3). It can be hypothesized that several *AHLs* modulate gene transcription, individually or as part of protein complexes in Arabidopsis and other species [7, 24, 47, 49]. Our studies did not show a direct biological mechanistic link between *AtAHL20* overexpression and repression of *FT* transcription. However, a close Clade-A *AHL* family member*, AtAHL22*, was shown to repress *FT* expression via a chromatin remodeling process [47]. This occurs via *FT* chromatin architecture modification through both H3 acetylation and methylation. In addition, Favero et al. [7] also showed that *AtAHL29*, a Clade-A *AHL*, directly binds to *YUC8* and *SAUR19* promoters resulting in gene expression repression. Lee and Seo, [24] went further and showed that *AtAHL27* and *AtAHL29* bind *YUC9* promoter and suppress gene expression via chromatin modification activities of SWI2/SNF2-RELATED 1 (SWR1) complex. Recently, Favero et al. [8] showed that AtAHL29 binds to PIF-targeted loci to reduce binding of PIF to these regions, thereby inhibiting transcriptional activation of growth promoting genes in Arabidopsis petioles. We hypothesize that AtAHL20 may also bind *FT* promoter elements and suppress its expression, perhaps individually or as part of complex. After all, AtAHL20 has already been shown to have binding affinities for several A/T-containing elements [10, 11]. A definitive answer to the question of the mechanism of gene repression may be provided via future studies that include yeast-one-hybrid and (chromatin immunoprecipitation) ChIP RT-qPCR experiments. Interestingly, *35S:AtAHL20* plants displayed similar adult plant phenotypes (dwarfism and late flowering) to *35S:AtAHL22* plants (Fig. 2), *esc-D/AtAHL27* as well as *sob3-D/AtAHL29* [35, 40]. Targeted Y2H studies showed that AtAHL20 interacted with AtAHL22, and SOB3/AtAHL29 (Fig. 5). It is, therefore, plausible that these AHLs function redundantly to regulate flowering time, possibly as part of a complex that includes AtAHL19, AtAHL20, AtAHL22, and AtAHL29.

### Missense mutation in the AT-hook domain abolishes *AtAHL20’s* overexpression phenotype

We hypothesized that a missense mutation in *AtAHL20’s* AT-hook domain would abolish function based on similar outcomes in other Clade-A *AHL* gene family members, *AtAHL29* [35] and *AtAHL22* [47]. Thus, it was not surprising that *35S:AtAHL20m* transgenic plants (Fig. 2a-f) lost the late-flowering phenotype typically observed when the wild-type *AtAHL20* gene is overexpressed. Our working hypothesis based on the works of [35, 47, 49] is that the second arginine residue in the AT-hook domain’s conserved R-G-R core is important for DNA binding, and without it, AtAHL20 would be unable to bind AT-rich DNA and recruit chromatin modifying components required to repress *FT* transcription. This is in line with a deletion mutant study from Lu et al., [28] who showed that removal of the entire AT-hook domain abolished AtAHL20’s suppression function. This raises an interesting question regarding the specific biological importance of the conserved R-G-R amino acid trio found in both type-1 and type-2 AT-hook motifs, versus peptide sequences flanking the AT-hook domain, for example. Would the mutation of the second arginine in the R-G-R core motif in all three AHL types (Type-I, −II, −III) also abolish overexpression phenotypes observed in transgenic plants overexpressing these genes? What role does the divergent nature of amino acid sequences flanking the R-G-R core play in Clade-A versus Clade-B AHLs? Studies in mammalian AHL orthologues, HMGA proteins, showed that the different types of AT-hook domains bind DNA with different affinities [5, 18]. HMGA proteins containing a type-1 AT-hook, similar to the one found in Clade-A AHLs (e.g. AtAHL20, AtAHL22, AtAHL27 and AtAHL29) [49], were found to confer the highest affinity to AT-rich DNA due to the nature of the peptide sequence adjacent to the R-G-R core motif. Interestingly, HMGAs containing a type-2 AT-hook, similar to one found in AtAHL6, have decreased DNA-binding affinity to AT-rich DNA [5, 18]. Notably, preliminary data from *AtAHL6* gain of function studies showed that transgenic plants overexpressing an aberrant gene carrying an R81- > H mutation (*35SAtAHL6m)* did not abolish the overexpression phenotype (early-flowering) observed in *35S:AtAHL6* plants. We can thus speculate whether this due to the fact that AtAHL6’s AT-hook domain has low DNA-binding affinity to begin with. In the future, it will be important to further investigate the effect of missense mutations in Clade-A versus Clade-B AHLs which contain different At-hook types, and whose conserved R-G-R core is flanked by divergent amino acid sequences.

### *FT* repression by *AtAHL20* negatively affects expression of downstream flowering pathway genes

Quantitative PCR data showed that overexpression of *AtAHL20* also resulted in repression of *TSF*, *AGL8* and *SPL3* expression (Fig. 4b). *AGL8* and *SPL3* function downstream of *FT* in the flowering pathway [16] whereas *TSF* acts redundantly with *FT* as a floral pathway integrator [44]. The fact that two redundant floral pathway integrators *FT* and *TSF* transcript levels are regulated in a similar manner in *35S:AtAHL20* transgenic plants, raises an interesting question. Does *AtAHL20* act directly on these two floral pathway integrators, or act upstream of them. Further experiments, including ChIP-Seq, yeast-one-hybrid assays would help identify AtAHL20’s direct targets. Previous work showed that overexpression of *AtAHL29*, a Clade-A Type-I gene (just like *AtAHL20*), also caused delayed flowering in Arabidopsis [35]. Interestingly, preliminary Chip-Seq data from our lab showed that AtAHL29 binds *FT.* Taken together, these data suggest that AtAHL20 may function in a similar manner, by directly binding to promoters of its downstream targets.

At the same time, it was interesting that overexpression of an aberrant AtAHL20 protein in *35S:AtAHL20m* transgenic plants only resulted in the elevation of *FT* transcript levels but not in downstream flowering pathway genes *TSF*, *AGL8* and *SPL3.* We speculate that *AtAHL20* indirectly affects expression of downstream targets via the direct repression of the main regulatory component of the flowering pathway, *FT*. Therefore, perhaps the elevation of *FT* transcript levels in *35S:AtAHL20m* transgenic plants is not of enough magnitude to dramatically alter the expression of downstream components.

## Conclusion

In conclusion, overexpression of *AtAHL20* repressed the expression of flowering pathway genes *FT, TSF, AGL8* and *SPL3*. In contrast, overexpression of an aberrant AtAHL20 protein harboring a missense mutation in the AT-hook domain abolished these phenotypes. These data suggest that *AtAHL20* is a transcription factor whose function is partly dependent on a conserved R-G-R core motif in the AT-hook domain.

## Methods

### Plant material

All *Arabidopsis thaliana* plants are in the Columbia *(Col-0)* background. *Col-0* and *AtAHL20* T-DNA insertion mutants, *ahl20-1* (Salk_144620) and *ahl20-2* (Salk_148971) seeds used in this study were obtained from the Arabidopsis Biological Resource Center (ABRC). Camelina plants *Camelina sativa* (L.) Crantz *var* Calena) were grown in a greenhouse (16 h light and 8 h dark) at 25 °C. Camelina seeds were provided by Dr. Scot Hulbert of Washington State University, who obtained them from Dr. Stephen Guy at Washington State University [15].

### Cloning and generation of transgenic Arabidopsis and Camelina plants

#### Arabidopsis thaliana

##### *AtAHL20* overexpression

Gateway compatible Entry vectors containing Arabidopsis *AHL* gene coding sequences and other genes used in this study were obtained from ABRC. To overexpress *AtAHL20*, Gateway Entry vector, pENTR223, was used in Gateway LR reactions (Invitrogen, Carlsbad, CA) with destination vector pEarlyGate100 binary vector (*35S* constitutive promoter) [6]. The binary vectors carrying *AtAHL20* cDNA were used to transform *Col-0* wild-type plants via the floral dip method [3]. To generate point mutations in AtAHL20’s AT-hook domain we used a QuikChange Lightning Site-Directed Mutagenesis Kit (Agilent, Santa Clara, CA) using Gateway compatible primers (Table 2). pENTR223 vector carrying *AtAHL20* cDNA was used as a template during the site-directed mutagenesis reaction. The resulting construct was sequenced to confirm the successful mutation of the arginine residues in the respective coding sequences.

**Table 2:** Primers used for cloning and gene expression studies in the study

Primer	Sequence
*Arabidopsis thaliana*	
AtAGL8qPCR-F	TGCGCTCCAGAAGAAGGATAAAGC
AtAGL8 qPCR-R	TTCCGTCAACGACGATGCACCA
AtAHL20CDS-F	ATGGCAAACCCTTGGTGGAC
AtAHL20CDS-R	TCAGTAAGGTGGTCTTGCGT
AtAHL20-ATTB-F	GGGGACAAGTTTGTACAAAAAAGCAGGCTTCATGGCAAACCCTTGGTGGAC
AtAHL20-ATTB-Rv	GGGGACCACTTTGTACAAGAAAGCTGGGTCGTAAGGTGGTCTTGCGTGGA
AtAHL20qPCR-Fw	CGTTGAGGTGGTCAACCGTA
AtAHL20qPCR-Rv	TTGCCTGCGTCTTGAGAAGT
AtFTqPCR-F	CCAAGTCCTAGCAACCCTCA
AtFTqPCR-R	TACACTGTTTGCCTGCCAAG
AtMDAR4q PCR-Fw	GCGGTGGCTATATCGGTATGG
AtMDAR4q PCR-Rv	AAAGAGACGTGCCATGCAGTG
AtSPL3qPCR-F1	CTTAGCTGGACACAACGAGAGAAGG
AtSPL3qPCR-R1	GAGAAACAGACAGAGACACAGAGGA
AtTSFqPCR-F	GAGTCCAAGCAACCCTCACCAA
AtTSFqPCR-R	CACCACAATACGATGAATTCCCGAG
UBQ10 -F	GGCCTTGTATAATCCCTGATGAATAAG
UBQ10 -R	AAAGAGATAACAGGAACGGAAACATAGT
Promoter-GUS	
AtAHL20Prom-ATTB-Fw	GGGGACAAGTTTGTACAAAAAAGCAGGCTTCTTGTAGCGGTAAATTGTGGCTTAA
AtAHL20Prom-ATTB-Rv	GGGGACCACTTTGTACAAGAAAGCTGGGTCGATTGACCAAAAACTGGAAATTCGC
*Camelina sativa*	
CsAHL20-Fw	AACGGTTTACTTAGCCGGGG
CsAHL20-Rv	GCAGCTATCACCATGACCGA
CsAHL20-ATTB-Fw	GGGGACAAGTTTGTACAAAAAAGCAGGCTATATGTCAAACCCTTGGTGGACG
CsAHL20-ATTB-Rv	GGGGACCACTTTGTACAAGAAAGCTGGGTCTCAGTATGGTGGTCGCGCGTG
CsFT-Fw	AGGAATTCACCGTGTCGTGA
CsFT-Rv	CGAGTGTTGAAGTTCTGGCG
CsMDAR4-Fw	TTGGCGAAATGAGGAGGCTT
CsMDAR4-Rv	AATGCCATGAGAAGGCGAA

##### GUS constructs

*AtAHL20’s* 1335 bp long promoter region was PCR amplified using Gateway-compatible primers (Table 2) and cloned into the Gateway compatible Entry vector pDONR221 via a BP reaction (Invitrogen, Carlsbad, CA). Following the BP reaction, the resultant Entry vector was sequenced to confirm the absence of mutations. pDONR221 Entry vectors carrying *AtAHL20* promoter were cloned into the Gateway-compatible destination vector pMDC163 (ABRC) via the Gateway LR reaction to generate a promoter: GUS expression binary vector.

Transgenic Arabidopsis plants expressing the above-mentioned constructs were generated in the wild type *Col-0* background via the floral-dip method [3]. Transgenic seeds were screened on 0.5× Linsmaier and Skoog modified basal medium supplemented with appropriate antibiotics containing 1.0% (w/v) phytagel (Sigma-Aldrich), 1.5% (w/v) sucrose and under continuous white light at 25 °C in a Percival E-30B growth chamber.

#### *Camelina sativa*

##### Overexpression of *CsAHL20*

*CsAHL20 (LOC104718987)* coding sequence was extracted from the NCBI database after a BLAST search using *AtAHL20 (AT4G14465)* sequence as a query. Primers (Table 2) were designed from the extracted sequence and were used to amplify *CsAHL20’s* coding sequence. The amplified PCR product was cloned into pDONR221 Entry vector via Gateway BP clonase II (Invitrogen, Carlsbad, CA) reaction to generate the pDONR221-CsAHL20 Entry vector. A Gateway LR clonase II (Invitrogen, Carlsbad, CA) reaction between pDONR221-CsAHL20 and the destination vector pUSH21 was performed to generate pUSH42-2. In this construct expression of *CsAHL20,* coding sequence and the selection marker DsRed were separately driven by CaMV 35S promoters. The binary vector was transformed into *Agrobacterium tumefaciens* strain GV3101 and used for plant transformation via the floral-dip protocol [27]. T_1_ seeds harvested from transformed plants were illuminated with a green LED light and fluorescent seeds were visually detected under a red filter [27]. Single insertion T-DNA T_2_ mutants were identified by screening for plants that produced 3:1 fluorescent: nonfluorescent seeds. Homozygous T_3_ pUSH42-2-CsAHL20 plants from single locus insertion lines were used for RT-qPCR analysis.

### Yeast-two-hybrid plasmids

A GAL4-based Y2H system was used in protein-protein interaction assays [38]. Yeast strain *L40ccU3,* bait vector (*pBTM116-GW-D9*) with *TRP1* reporter marker and prey vector (*pACT2-GW*) with *LEU* reporter marker were obtained from Dr. Hanjo Hellmann’s lab (Washington State University, Pullman, WA). Gateway Entry vectors carrying *AtAHL6, AtAHL19, AtAHL20, AtAHL22* and *AtAHL29’s* coding sequences genes were used in LR reactions to clone the respective open reading frames into the bait and prey vectors (*pBTM116-GW-D9*) and (*pACT2-GW*), respectively. Competent yeast cells were transformed with bait and prey plasmid constructs using a standard lithium acetate protocol. Transformed yeast competent cells were incubated for three days at 28 °C on SD minimal medium supplemented with Leu and His (SDII). Four randomly selected colonies were diluted 1:2000 in autoclaved distilled water before 20 μL were simultaneously dropped on both SDII and SDIV lacking tryptophan, leucine, histidine and uracil and containing predetermined levels of 3-amino-1, 2, 4-triazol (3-AT). Yeast was incubated at 28 °C for 3-6 days.

### RNA extraction, cDNA synthesis, RT-qPCR, semi-quantitative PCR and data analysis

Total RNA was extracted from 10-day old Camelina seedlings grown on ½ × MS medium using Plant RNA mini kit (Qiagen, Valencia, CA) according to manufacturer’s recommendations. For Arabidopsis, total RNA was extracted from rosette leaves collected from 21-day old adult plants (analyzed to quantify *FT*, *TSF*, *AGL8* and *SPL3* via RT-qPCR), as well as from 7-day old seedling roots, whole 7-day old seedling, adult plant rosette leaf, 7-day old seedling hypocotyls, flowers and siliques (used for semi-quantitative PCR for *AtAHL20* tissue specific expression). On-column DNAse treatment was performed to digest any potential contaminating genomic DNA. Complementary DNA (cDNA) was synthesized from total RNA (500 ng) using the iScript Reverse Transcription Super mix (Bio-Rad, Hercules, CA). RT-qPCR was carried out using Bio-Rad’s SSO Advanced Universal SYBR Green Super Mix (Bio-Rad, Hercules, CA) and 10-fold diluted cDNA templates (synthesized above) on a Bio-Rad’s CFX96 Touch Real-Time PCR Detection System. Melting curves of SYBR green wells were cross checked to eliminate nonspecific amplification. Data are normalized to MDAR4 messenger ribonucleic acid (mRNA) expression (internal control), and fold changes are displayed relative to control plant lines. Error bars represent standard deviations of technical replicates (*n* = 3). Three biological replicates were used from each plant line.

### RNA-Seq library preparation

Total RNA was extracted from rosette leaves harvested from 21-day old growth-chamber-grown plants using MagJET Plant RNA Purification Kit (Thermo Fisher Scientific, Waltham, MA). The Dynabeads mRNA DIRECT Kit (Thermo Fisher Scientific, Waltham, MA) was used for purification of intact polyadenylated (polyA) mRNA. RNA-Seq libraries were prepared using the Ion Total RNA-Seq Kit v2 (Life Technologies, Carlsbad, CA) following the manufacturer’s protocol.

RNA-Seq datasets were analyzed using CLC Genomics Workbench software (Qiagen, Valencia, CA). RNA-Seq libraries were constructed from RNA extracted from rosette leaf tissue pooled from three independent plants. Following Kal’s Z-test [22], genes were classified as differentially expressed with a False Discovery Rate (FDR) adjusted *p*-value < 0.05 and a fold-change absolute value > 3.

### Read mapping and differential expression

Reads which already had adaptor sequences removed by the Torrent Suite ver 4.2.1 sequencing software (Thermo Fisher Scientific, Waltham, MA), were quality trimmed using the default setting in CLC Genomics Workbench 7.5 (Qiagen, Valencia, CA). After preprocessing the RNA-Seq data, the reads were mapped to the TAIR10 version of the Arabidopsis genome using CLC Genomics Workbench 7.5 (Qiagen, Valencia, CA). Read counts for each gene were quantified using the RNA-Seq Analysis tool using the default settings. Differential expression of original values was determined with the proportions statistical analysis tool, using Kal’s Z-test with FDR correction.

### Histochemical GUS analysis

GUS analysis was performed as described by [48] on six-day old seedlings, 12-day old plants, and floral structures from flowering plants grown in the greenhouse.

### Flowering time analysis

It has been observed that transplanting seedlings to soil can cause stresses that can alter flowering time. Consequently, all seeds were directly sown in pots containing a pre-watered soil mix Sunshine 50 Mix4 (Aggregate) LA4, (Green Island Distributers Inc.; Riverhead, N.Y). These pots were subsequently incubated in darkness for 7 days at 4 °C to induce near-uniform germination. After that, pots were transferred to growth chambers under the following conditions: white light (200 μmol m^− 2^ s^− 1^), 21 °C and 60-70% humidity. Once the seedlings were several days old, they were thinned to one per pot by clipping using small scissors. Experience in the lab suggests that removal of whole seedlings causes root damage to neighboring seedlings, which in turn can cause damage/stress that can potentially lead to altered flowering time. This approach gives the most uniform and repeatable flowering time results for each genotype. To measure flowering time, we counted the number of days from germination time until the floral stem was 0.5 cm above the basal rosette.

### Sequence alignment

AtAHL20, AtFT, CsAHL20 and CsFT nucleotide and protein sequences were downloaded from NCBI database (Data S3). Both nucleotide and protein sequence alignment were aligned using BOXSHADE public server https://embnet.vital-it.ch/software/BOX_form.html.

## Supplementary Information

**Additional file 1:** **Data S1.** RNA-Seq data of genes down-regulated in *35S:AtAHL20* versus the *Col-0* wild type. **Data S2.** RNA-Seq data of genes up-regulated in *35S:AtAHL20* versus the *Col-0* wild type.

**Additional file 2:** **Data S3.** *AtAHL20*, *CsAHL20*, *AtFT* and *CsFT* nucleotide and protein sequences used in cloning of overexpression vectors and in sequence alignments.

## Data Availability

The RNA-Seq dataset(s) supporting the conclusions of this article is (are) available in the NCBI Sequence Read Archive (SRA) repository, accession number PRJNA671767 under the following link: https://www.ncbi.nlm.nih.gov/Traces/study/?acc=PRJNA671767 The datasets used and/or analyzed during the current study are available from the corresponding author on reasonable request.

## Abbreviations

3-AT: 3-amino-1,2,4-triazole
ABRC: Arabidopsis Biological Resource Center
AGL8: AGAMOUS-LIKE 8
AHL: AT-HOOK MOTIF CONTAINING NUCLEAR LOCALIZED
AHL20: AT-HOOK MOTIF CONTAINING NUCLEAR LOCALIZED #20
ANOVA: Analysis of variance
AT: Adenine Thymine
At: *Arabidopsis thaliana*
CaMV: Cauliflower mosaic virus
*Col-0*: Columbia
Cs: *Camelina sativa*
DNA: Deoxyribonucleic acid
FT: FLOWERING LOCUS T
FDR: False discovery rate
GO: Gene ontology
GUS: *βeta*-glucuronidase
HMG: HIGH MOBILITY GROUP
LD: Long days
SD: Short days
MDAR4: MONODEHYDROASCORBATE REDUCTASE 4
mRNA: Messenger Ribonucleic acid
NCBI: National Center for Biotechnology Information
PPC: PLANT AND PROKARYOTE CONSERVED
R-G-R-P: Arginine-glycine-arginine-proline
RNA-seq: Ribonucleic acid sequencing
RT-qPCR: Reverse transcription quantitative polymerase chain reaction
SD: Synthetic defined
SEM: Standard error of mean
SPL3: SQUAMOSA PROMOTER BINDING PROTEIN-LIKE 3
T-DNA: Transfer deoxyribonucleic acid
TF: Transcription factor
TSF: TWIN SISTER OF FT
