# Supplementary material for: Overexpression of AtAHL20 causes delayed flowering in Arabidopsis via repression of FT expression
Source: BMC Plant Biol. 2020 Dec 11;20:559. doi: 10.1186/s12870-020-02733-5 (PMC7731500; doi:10.1186/s12870-020-02733-5)
Supplement: Supplementary file 2 — Additional file 2: Data S3. AtAHL20, CsAHL20, AtFT and CsFT nucleotide and protein sequences used in cloning of overexpression vectors and in sequence alignments. [file 12870_2020_2733_MOESM2_ESM.docx]

>AtAHL20(AT4G14465)_CDS

ATGGCAAACCCTTGGTGGACGAACCAGAGTGGTTTAGCGGGCATGGTGGACCATTCGGTCTCCTCAGGCCATCACCAAAACCATCACCACCAAAGTCTTCTTACCAAAGGAGATCTTGGAATAGCCATGAATCAGAGCCAAGACAACGACCAAGACGAAGAAGATGATCCTAGAGAAGGAGCCGTTGAGGTGGTCAACCGTAGACCAAGAGGTAGACCACCAGGATCCAAAAACAAACCCAAAGCTCCAATCTTTGTGACAAGAGACAGCCCCAACGCACTCCGTAGCCATGTCTTGGAGATCTCCGACGGCAGTGACGTCGCCGACACAATCGCTCACTTCTCAAGACGCAGGCAACGCGGCGTTTGCGTTCTCAGCGGGACAGGCTCAGTCGCTAACGTCACCCTCCGCCAAGCCGCCGCACCAGGAGGTGTGGTCTCTCTCCAAGGCAGGTTTGAAATCTTATCTTTAACCGGTGCTTTCCTCCCTGGACCTTCCCCACCCGGGTCAACCGGTTTAACGGTTTACTTAGCCGGGGTCCAGGGTCAGGTCGTTGGAGGTAGCGTTGTAGGCCCACTCTTAGCCATAGGGTCGGTCATGGTGATTGCTGCTACTTTCTCTAACGCTACTTATGAGAGATTGCCCATGGAAGAAGAGGAAGACGGTGGCGGCTCAAGACAGATTCACGGAGGCGGTGACTCACCGCCCAGAATCGGTAGTAACCTGCCTGATCTATCAGGGATGGCCGGGCCAGGCTACAATATGCCGCCGCATCTGATTCCAAATGGGGCTGGTCAGCTAGGGCACGAACCATATACATGGGTCCACGCAAGACCACCTTACTGA

>CsAHL20(LOC104718987)_CDS

ATGTCAAACCCTTGGTGGACGAACCAGAGTGGTTTAGCCGGTATGGTAGACCACTCAGCCTCCTCCGGCTACCACCAAAACCATCATCACCAAAGTCTTCTTAGCAAAGGAGATCTTGGAATAGCCATGAATCAGAGCCAAGACAACGACCAAGATGAAGAAGACGACCCTAGGGAAGGAGCAGTTGAGGTGGTCAACCGTAGACCAAGAGGAAGACCACCAGGATCAAAAAACAAACCTAAACCTCCAATCTTTGTGACAAGAGACAGCCCCAACGCACTCCGTAGCCATGTCCTGGAGATCTCCGACGGCAGCGACGTTGCGGACACAATCGCTCACTTCTCCAGACGCAGACAACGCGGCGTTTGTGTTCTCAGCGGCACAGGCTCAGTCGCTAACGTCACCCTCCGTCAAGCTGCCACACCAGGAGGTGTAGTCTCTCTCCAAGGCAGGTTTGAGATCTTATCCTTAACCGGTGCTTTCCTCCCTGGACCTTCCCCACCCGGGTCAACCGGTTTAACGGTTTACTTAGCCGGGGTTCAGGGTCGAGTCGTTGGGGGTAGCGTCGTGGGACCGCTCCTGGCCATAGGGTCGGTCATGGTGATTGCTGCTACTTTCTCTAACGCTACTTATGAGAGATTGCCCATGGAAGAAGAGGAAGACGGTGGAGGCTCAAGACCGATTCATGGAGGTGGTGACTCGCCACCCGGAATGGGTAGTAGCCTGCCTGATCCATCTGTGATGGCCGGGTCAGGGCCAGGTTACAATACGCCGCCGCATCTGATTCCGAATGGGGCTGGTCAGTTAGGGCACGAACCATATACATGGGTCCACGCGCGACCACCATACTGA

>AtAHL20(AT4G14465)_protein

MANPWWTNQSGLAGMVDHSVSSGHHQNHHHQSLLTKGDLGIAMNQSQDNDQDEEDDPREGAVEVVNRRPRGRPPGSKNKPKAPIFVTRDSPNALRSHVLEISDGSDVADTIAHFSRRRQRGVCVLSGTGSVANVTLRQAAAPGGVVSLQGRFEILSLTGAFLPGPSPPGSTGLTVYLAGVQGQVVGGSVVGPLLAIGSVMVIAATFSNATYERLPMEEEEDGGGSRQIHGGGDSPPRIGSNLPDLSGMAGPGYNMPPHLIPNGAGQLGHEPYTWVHARPPY

> CsAHL20(LOC104718987)_protein

MSNPWWTNQSGLAGMVDHSASSGYHQNHHHQSLLSKGDLGIAMNQSQDNDQDEEDDPREGAVEVVNRRPRGRPPGSKNKPKPPIFVTRDSPNALRSHVLEISDGSDVADTIAHFSRRRQRGVCVLSGTGSVANVTLRQAATPGGVVSLQGRFEILSLTGAFLPGPSPPGSTGLTVYLAGVQGQVVGGSVVGPLLAIGSVMVIAATFSNATYERLPMEEEEDGGGSRPIHGGGDSPPGMGSSLPDPSVMAGSGPGYNMPPHLIPNGAGQLGHEPYTWVHARPPY

>AtFT(AT1G65480)_CDS

ATGTCTATAAATATAAGAGACCCTCTTATAGTAAGCAGAGTTGTTGGAGACGTTCTTGATCCGTTTAATAGATCAATCACTCTAAAGGTTACTTATGGCCAAAGAGAGGTGACTAATGGCTTGGATCTAAGGCCTTCTCAGGTTCAAAACAAGCCAAGAGTTGAGATTGGTGGAGAAGACCTCAGGAACTTCTATACTTTGGTTATGGTGGATCCAGATGTTCCAAGTCCTAGCAACCCTCACCTCCGAGAATATCTCCATTGGTTGGTGACTGATATCCCTGCTACAACTGGAACAACCTTTGGCAATGAGATTGTGTGTTACGAAAATCCAAGTCCCACTGCAGGAATTCATCGTGTCGTGTTTATATTGTTTCGACAGCTTGGCAGGCAAACAGTGTATGCACCAGGGTGGCGCCAGAACTTCAACACTCGCGAGTTTGCTGAGATCTACAATCTCGGCCTTCCCGTGGCCGCAGTTTTCTACAATTGTCAGAGGGAGAGTGGCTGCGGAGGAAGAAGACTTTAG

>CsFT(LOC104750330)_CDS

ATGTCTACAACCGTGCGAGACCCTCTTACAGTAAGCCGAGTTGTTGGAGACGTTCTTGATCCGTTCAATAGATCGATCTCTTTAAGGGTTACTTATGGCCAAAGAGAGGTGACTAATGGCTTGGATCTAAGGCCTTCTCAAGTTCAGAACAAACCAAGAGTTGAGATTGGTGGAGAAGACCTCAGGAACTTCTACACTTTGGTCATGGTGGATCCAGATGTCCCAAGTCCTAGCAACCCTCATCTCCGAGAATATCTCCACTGGTTGGTGACTGATATCCCTGCTACAACTGGAACATCCTTTGGAAATGAGATTGTGTGTTACGAAAATCCAAGTCCTTCCGCAGGAATTCACCGTGTCGTGATGATATTGTTTCGGCAGCTTGGGAGGCAAACAGTGTACGCACCAGGGTGGCGCCAGAACTTCAACACTCGCGAATTTGCTGAGATCTACAATCTCGGCCTTCCCGTGGCCGCAGTTTTCTTCAATTGTCAGAGGGAGAGTGGTTGCGGAGGAAGAAGAACTTAG

>AtFT(AT1G65480)_protein

MSINIRDPLIVSRVVGDVLDPFNRSITLKVTYGQREVTNGLDLRPSQVQNKPRVEIGGEDLRNFYTLVMVDPDVPSPSNPHLREYLHWLVTDIPATTGTTFGNEIVCYENPSPTAGIHRVVFILFRQLGRQTVYAPGWRQNFNTREFAEIYNLGLPVAAVFYNCQRESGCGGRRL

>CsFT(LOC104750330)_protein

MSTTVRDPLTVSRVVGDVLDPFNRSISLRVTYGQREVTNGLDLRPSQVQNKPRVEIGGEDLRNFYTLVMV

DPDVPSPSNPHLREYLHWLVTDIPATTGTSFGNEIVCYENPSPSAGIHRVVMILFRQLGRQTVYAPGWRQ

NFNTREFAEIYNLGLPVAAVFFNCQRESGCGGRRT

**Data S3.** *AtAHL20*, *CsAHL20*, *AtFT* and *CsFT* nucleotide and protein sequences used in cloning of overexpression vectors and in sequence alignments.
